# Antigenic drift and epidemiological severity of seasonal influenza in Canada

**DOI:** 10.1038/s41598-022-19996-7

**Published:** 2022-09-17

**Authors:** Zishu Chen, Christina Bancej, Liza Lee, David Champredon

**Affiliations:** 1 National Microbiology Laboratory, Public Health Risk Sciences Division, Public Health Agency of Canada, Guelph, ON Canada; 2 Surveillance and Epidemiology Division, Centre for Immunization and Respiratory Infectious Disease, Public Health Agency of Canada, Ottawa, ON Canada; 3 Dalla Lana School of Public Health, University of Toronto, Toronto, ON Canada

**Keywords:** Computational biology and bioinformatics, Evolution, Influenza virus

## Abstract

Seasonal influenza epidemics circulate globally every year with varying levels of severity. One of the major drivers of this seasonal variation is thought to be the antigenic drift of influenza viruses, resulting from the accumulation of mutations in viral surface proteins. In this study, we aimed to investigate the association between the genetic drift of seasonal influenza viruses (A/H1N1, A/H3N2 and B) and the epidemiological severity of seasonal epidemics within a Canadian context. We obtained hemagglutinin protein sequences collected in Canada between the 2006/2007 and 2019/2020 flu seasons from GISAID and calculated Hamming distances in a sequence-based approach to estimating inter-seasonal antigenic differences. We also gathered epidemiological data on cases, hospitalizations and deaths from national surveillance systems and other official sources, as well as vaccine effectiveness estimates to address potential effect modification. These aggregate measures of disease severity were integrated into a single seasonal severity index. We performed linear regressions of our severity index with respect to the inter-seasonal antigenic distances, controlling for vaccine effectiveness. We did not find any evidence of a statistical relationship between antigenic distance and seasonal influenza severity in Canada. Future studies may need to account for additional factors, such as co-circulation of other respiratory pathogens, population imprinting, cohort effects and environmental parameters, which may drive seasonal influenza severity.

## Introduction

Seasonal influenza epidemics occur globally every year, leading to a high burden of mortality and morbidity, especially in children, older adults, and individuals with underlying health conditions. In Canada, it has been estimated that approximately 12,200 hospitalizations^1^ and 3500 deaths^2^ are attributed to seasonal influenza epidemics on average each year. Epidemic severity varies from season to season. Although epidemic influenza dynamics are complex and not well-understood, one of the major drivers of this seasonal variation is posited to be the antigenic drift of influenza viruses^3^, resulting from the accumulation of mutations from one season to another. In particular, mutations in the hemagglutinin protein, which contains antigenic sites targeted by neutralizing antibodies, can lead to immune escape and may also have implications for vaccine effectiveness^4,5^. Traditionally, antigenic differences between viruses have been determined experimentally using laboratory procedures such as the hemagglutination inhibition assay; however, these methods are time-consuming and costly. More recently, methods based on changes to genetic sequences have been explored as an alternative approach to antigenic cartography^6–8^. Thanks to the sequencing efforts shared publicly by many participating laboratories worldwide, it is possible to infer the antigenic drift of circulating influenza viruses across many seasons using these computational methods. Previous studies have integrated sequence-based antigenic distances and epidemiological measures of severity obtained through surveillance data to link antigenic drift to seasonal severity^9,10^. However, to our knowledge, no studies have measured this association within a Canadian context. In this study, we aim to assess if a statistical relationship can be detected between the antigenic drift of seasonal influenza and the epidemiological severity of seasonal epidemics. We limit our analysis to Canada.

## Methods

### Genetic data

Protein sequence data for the HA segment of all circulating A/H1N1 (seasonal and 2009 pandemic), A/H3N2, and B strains collected from human hosts between 2006-08-01 and 2020-08-01 were downloaded from GISAID. We did not restrict the sequence to the predominant strain(s) during this initial data collection because we aimed to be as exhaustive as possible, in case some circulating strains sequenced were not identified as antigenically relevant. The following metadata were extracted from the FASTA header of each sequence: strain name, collection date, location (obtained from the strain name), and GISAID accession number. Only viruses collected in Canada were included for analysis (see table in supplementary file S1 for the complete list of sequence accession numbers).

**Figure 1:**
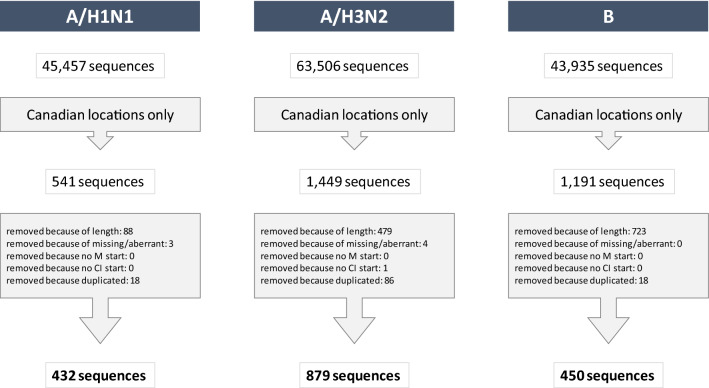
Flowchart for the cleaning process of the genetic sequences for influenza A/H1N1, A/H3N2 and B. The number of sequences at the top represents the total number of sequences downloaded from GISAID, filtering the virus type or subtype only. The number at the bottom represents the final number of sequences used in this analysis.

Sequence clean-up was performed. We excluded HA sequences that: did not start with methionine (“M”); did not end with “CI”; had a length different from 565–566 amino acids (type A) or 582–587 amino acids (type B); contained more than 0.5% of aberrant amino acids. The list of sequences was deduplicated based on the strain name^8^. After cleaning, there were 432 H1N1, 879 H3N2 and 450 B sequences in total. Figure 1 illustrates this filtering process. The MUSCLE program^11^ was used for sequence alignment using the parameterization “-maxiters 1—diags—sv—distance1 kbits20_3”.

Since Canada’s flu season is in line with temperate zone Northern hemisphere influenza seasonality (generally starting in the fall of one calendar year and ending in the spring of the following calendar year), sequences were divided by season based on their sample collection date. Samples that were collected prior to week 35 of the calendar year were considered to be from the flu season starting in the previous calendar year. For example, a sequence collected on Feb. 1, 2015 would count as part of the circulating strains from the 2014/2015 flu season.

### Epitope determination and antigenic distance calculation

We performed a literature review to identify antigenically relevant epitopes of the HA protein for influenza A/H1N1, A/H3N2 and B.

Through experimental methods such as monoclonal antibody selection of escape mutants or X-ray crystallography, epitope mapping has led to the establishment of major or “canonical” antigenic sites on the HA protein and other surface proteins, such as neuraminidase. However, many other epitopes have been identified outside of these canonical sites as well^12,13^. Due to the suggestion that epitopes can have “fuzzy” boundaries^14^ and may also change over time as the virus-host relationship evolves^15^, we considered multiple epitope formulations in our analysis.

Hence, we defined the sequence-based antigenic distance between two aligned sequences as the Hamming distance between their respective HA proteins, considering only the amino acids at the antigenic sites. Moreover, to reflect the uncertainty about the actual epitope positions, we created four definitions of the antigenic distance: (1) a “narrow” definition, comprising only canonical antigenic sites—Sa, Sb, Ca1, Ca2 and Cb for H1N1; A, B, C, D and E for H3N2^16^; and the 120 loop, 150 loop, 160 loop, 190 helix, and 230 region for B^17–19^; (2) a “broad” definition, including a buffer of three amino acids before and after each canonical antigenic site; (3) the full sequence of the HA1 domain, which contains the major antigenic sites; and (4) the full sequence of the HA protein (including both HA1 and HA2 domains). As the results from the HA1 and HA1 + HA2 sequences were similar in preliminary analyses, we only considered the full HA protein sequence containing both HA1 and HA2 (referred to as “full”) moving forward. Furthermore, to test the robustness of these baseline assumptions, we also defined alternative epitope positions to run our analysis. The full list of residue positions is given in Supplementary file S2. We calculated the inter-season antigenic distance independently for A/H1N1, A/H3N2 and B by taking the mean of pairwise distances between all sequences of a season and its preceding season.

### Epidemiologic data

To quantify the severity of a seasonal epidemic for a given influenza virus subtype, we constructed a “severity index” (defined below), composed of multiple metrics from various epidemiological data sources.

Laboratory-confirmed influenza case numbers from the 2007/2008 to 2019/2020 seasons were obtained from the Respiratory Virus Detection Surveillance System at the Public Health Agency of Canada, which collects data on the number of influenza tests and positive detections across Canada and releases weekly reports ^20^. Using this data, positivity rates and seasonal peak positivity were determined.

Influenza and pneumonia-associated deaths in Canada were obtained from a dataset on leading causes of death from 2000 to 2020 derived from Canadian Vital Statistics death data^21^. Death counts were normalized by the population of Canada of the corresponding year^22^. The death rate of year Y was associated with the (Y − 1)/Y seasonal epidemic (i.e., deaths recorded in 2012 were associated with the 2011/2012 season). Pediatric hospitalization data was available to FluWatch, Canada’s national influenza surveillance program, through the IMPACT pediatric hospital-based surveillance network^23^. The data consisted of a line list with hospitalization date and patient age. The counts were normalized by the population of the 5 year age group corresponding to the patient’s age and the associated flu season was assigned based on the hospitalization date being before or after week 35.

The basic reproduction number (R_0_) was calculated at the start of each season using the approximation outlined by Park et al.^24^ on the number of laboratory-confirmed cases. The slope of the log-transformed incidence curve was calculated using data points from 10 to 3 weeks before the seasonal peak and taken as the rate of spread for each season. Based on the literature, we set the mean generation interval to 3.6 days and its standard deviation to 1.6 days^25^.

The parameter R_0_ was calculated only for the seasons where influenza types and subtypes were circulating with substantial prevalence. For H1N1, these were the 2013/2014, 2015/2016, 2018/2019, 2019/2020 seasons; for H3N2, only the 2010/2011 to 2012/2013, 2014/2015, and 2016/2017 to 2018/2019 seasons were included; for B, all seasons except for 2009/2010.The time series of reported influenza cases in Canada are shown in Supplementary file S3.

### Immunization data

Influenza vaccine coverage data was obtained from two sources: the Seasonal Influenza Vaccination Coverage Survey (SIVC) and the annual component of the Canadian Community Health Survey (CCHS). SIVC data was available for adults aged 18 years and older, as well as stratified by age group and chronic medical condition, from 2015/2016 to 2020/2021. Population-wide and age-specific CCHS data was available for individuals aged 12 years and older from 2015 to 2020. Since the outcome measured was “Influenza immunization in the past 12 months,” this corresponded approximately to the 2014/2015 to 2019/2020 flu seasons. Vaccine effectiveness estimates by influenza type and subtype were available from the Canadian Sentinel Practitioner Surveillance Network for the 2004/2005 to 2019/2020 seasons^26^. After retrieving the data, we observed that vaccine coverage data was missing for several seasons during the study period and did not vary greatly in the seasons we had data for. Hence, we assumed vaccine coverage to be constant and excluded it from the statistical analysis.

### Severity index

The severity of seasonal influenza epidemics is multi-faceted. Large numbers of symptomatic cases can have a significant societal and economic impact, with an estimated average cost of $14,000 per hospitalization^27^ and approximately 14 work hours lost per employee per seasonal influenza infection^28^. Influenza-associated hospitalizations can stress health care systems, especially during times of peak flu activity, reducing the overall quality of care (e.g., cancelled elective surgeries, staffing changes, etc.)^29^. High transmission rates also contribute to severity as cases seek care in a short period of time, placing further burden on health systems. In examining the severity of an influenza season, many studies consider one metric at a time (e.g., case fatality ratio^30^, peak excess pneumonia and influenza-associated mortality rates^31^, etc.). Others have developed indices that utilize measures along a single dimension^32^ or that provide qualitative, but not quantitative, assessments of seasonal severity^33,34^. Based on previous work done to integrate multiple measures into a composite indicator of severity^35^, we constructed a “severity index” to quantify the severity of a given influenza season independently for influenza A/H1N1, A/H3N2 and B. For a given season, we defined $$p$$, the peak positivity calculated from reported influenza cases whose (sub)type has been laboratory-confirmed; $$h$$, the number of hospitalizations associated with influenza; $$d$$, the number of deaths per 100,000 associated with influenza and pneumonia; $${R}_{0}$$, the basic reproduction number (estimated as described above). Then, we defined the severity index $$S$$ as:

$$S \sim \beta_{1} *a + \beta_{2} *v + \beta_{3} *a*v,$$

where $P = \left( {{\text{logit}}\left( p \right) - m_{p} } \right)/\sigma_{p}$, $H = \left( {\log \left( h \right) - m_{h} } \right)/\sigma_{h}$, $D = \left( {\log \left( d \right) - m_{d} } \right)/\sigma_{d}$ and $R = \left( {R_{0} - m_{{R_{0} }} } \right)/\sigma_{{R_{0} }}$. The parameter $m_{x}$ is the mean of log(x) (or logit(x) when *x* represents the peak positivity) across all seasons available from our dataset, and $\sigma_{x}$ its standard deviation. The parameter $n$ is the number of variables (among $P,H,D,R$) available for a given season ($1 \le n \le 4)$. Supplementary file S4 is a table detailing the values of the components that constitute the severity index.

### Statistical analysis

To assess the relationship between the severity index and the inter-seasonal antigenic distance, we performed the following linear regression:

$$S \sim \beta_{1} *a + \beta_{2} *v + \beta_{3} *a*v$$

where $S$ is the severity index, $a$ is the mean inter-season antigenic distance, and $v$ is the vaccine effectiveness, for a given influenza virus (i.e., A/H1N1, A/H3N2 and B) and season.

We imposed a data quality constraint on the regression, that is, the regression was performed only on data points that had a mean inter-season antigenic distance calculated with at least 20 paired sequences and a severity index calculated with not more than one variable missing (n >  = 3). This was applied irrespective of the sequence-based antigenic distance definition used (i.e., “narrow”, “broad” or “full”). In addition, seasons with missing vaccine effectiveness estimates were excluded from the analysis.

To account for the uncertainty of the sequence-based antigenic distance, we repeated the linear regression using the following Monte Carlo algorithm. The inter-season antigenic distance was assumed to be normally distributed with mean and standard deviation parameters informed by their empirical values. We chose to use a normal distribution instead of the empirical distribution (bootstrap method) to avoid biases induced by small sample sizes. Then, a distance value was sampled from this normal distribution for each season. Finally, the regression was run on those sampled points and the regression coefficients as well as their associated *p*-values were recorded. This procedure was repeated 100 times. The inference about the relationship between severity and antigenic distance was assessed by considering the distribution of the coefficients and *p*-values recorded across the 100 iterations.

All analyses were conducted using R version 4.1. The code used to conduct the full analysis is available at https://github.com/phac-nml-phrsd/publication-2022-flu-drift

## Results

**Figure 2:**
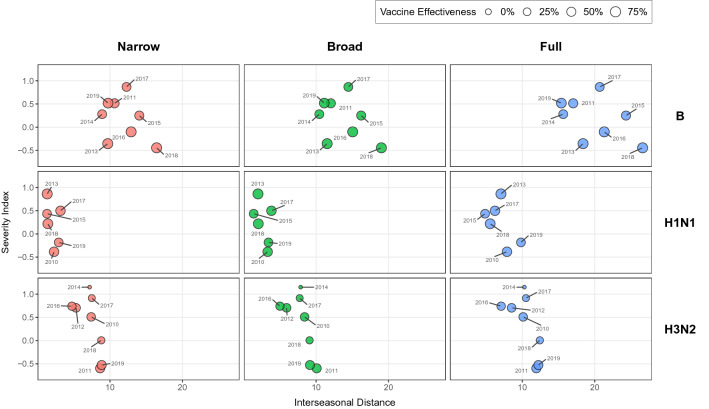
Relationship between the epidemiological severity (as measured by the Severity Index) and the genetic drift of influenza (as measured by the mean Hamming distance between a season and the previous one). Each row represents a type/subtype and each column a different definition of the antigenic distance. The label next to each point indicates the starting year of the epidemic season (e.g., 2014 for the 2014/2015 season). The size of the point is proportional to the vaccine effectiveness observed that year for a given type/subtype.

The data points for influenza A/H1N1, A/H3N2 and B are shown in Fig. 2. The data quality constraints gave a total of seven data points (i.e., seasons) for A/H1N1, eight for A/H3N2 and seven for B.

**Figure 3:**
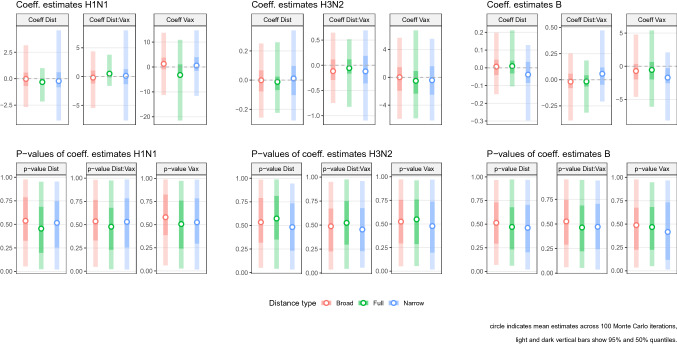
Regression results. The top panels represent the estimated values of the regression coefficients and the bottom panels their corresponding *p*-values. In all panels, the circle represents the mean estimate, the light and dark vertical bars 95% and 50% quantiles across 100 Monte Carlo iterations. “Dist” represents the covariate for the mean inter-season antigenic distance; “Vax” the covariate for the vaccine effectiveness; “Dist:Vax” the interaction coefficient.

The outputs from the linear regressions show that we were not able to detect any statistically significant relationship between the epidemiological severity and the inter-season antigenic distance for any of the three viruses considered in our study. The distributions of the regression coefficients are shown in Fig. 3 and their values presented in Supplementary file S5. The top row shows that estimates of mean values for the slope coefficients are close to zero. The bottom row of Fig. 3 shows the distribution of the associated *p*-values, which all indicate a lack of statistical significance. Similar results were observed across the different antigenic distance definitions used.

To assess the robustness of these results, we performed the same analysis after calculating the antigenic distance with a lag of two seasons instead of one (i.e., determining mean pairwise distances between a given season and the season two years prior). We also simplified the severity index, using alternatively only the peak positivity rate, pediatric hospitalization rate, mortality rate, or the estimate of the basic reproduction number. We obtained similar results in each case (Supplementary files S6 and S7). Moreover, results from sensitivity analyses using alternatively-defined epitope positions (Supplementary file S2) were analogous to the regression results using the baseline definitions (Supplementary file S8).

## Discussion

There are probably many factors affecting the severity of an influenza season, including viral genetic drift, the immune history of the population, the prevalence of other seasonal respiratory pathogens, climatic factors, and the effectiveness and coverage of influenza vaccines. Influenza seasons are usually made of “sub-epidemics” from three influenza lineages (A/H1N1, A/H3N2 and B), making attempts to better understand seasonal influenza epidemics even more challenging. This seasonal influenza system, affected by factors interacting in complex ways, has persistently evaded our understanding of its epidemiological impact on populations.

The ecological analysis presented here attempted to tackle seasonal influenza in a simplified approach to identify potential main effects of antigenic distance on epidemic severity. Our hypothesis was that the severity of a given seasonal influenza season is driven by the antigenic drift of circulating influenza viruses and the effectiveness of seasonally updated influenza vaccines. To further limit the complexity, we focused on a single geographical location, Canada, which has an established national influenza surveillance system.

Even a simplified approach has intrinsic limitations, starting with the observation metrics of seasonal epidemics of influenza. First, it is still not clear how to define the genetic drift of influenza viruses. Antigenic sites, regions on the viral genome prone to mutations that impact immune response, have been broadly identified since the 1980s^36,37^. However, a precise mapping of the potential impact of influenza virus epitopes is still missing and may never be fully established, given that these epitopes are defined by their interactions with a constantly evolving and heterogeneous human immune system. Viral mutations not only influence the immune response, but also the patient-level severity of the disease. In fact, even single amino acid mutations in the HA protein can have an outsized effect on antibody recognition^38^, and one mutation in particular has been associated with an increased risk of severe or fatal disease in influenza A H1N1pdm09 infections^39^. Genetic drift in the NA surface protein, as well as in the internal genes, is also known to play a role in virulence.

In addition to the difficulties of nano-scale metrics for genetic mutations, observing macro-scale metrics that translate the epidemiological severity of an influenza season in a population is challenging. Only severe influenza cases are usually identified with a (gold-standard) molecular test, biasing observations. Influenza-associated hospitalizations probe a small segment of the population but may be more representative of severity if records in health systems can identify influenza-related illness as the cause for hospitalization. Influenza-associated mortality is also usually difficult to assess because recording of the actual cause of death may not always be accurate. Furthermore, linkage to the viral type and subtype, or even viral testing—essential when performing an epidemiological analysis—is often lacking for hospitalization and mortality data.

Volume and consistency of historical data is also a challenge. Despite having circulated in human populations for centuries (with microbiological evidence of influenza infection dating back to at least 1918^40^) and likely for millennia (with influenza-like illness (ILI) reported as far back as ancient Greece and the first ILI epidemic recorded in 1173^41^), data collection related to influenza infections may not be consistent in time and space within a jurisdiction. The longest time-series available, spanning multiple decades, are often those of ILIs^42,43^ as sequencing has only become more affordable in the last decade or so^44^. Unfortunately, ILI data are not specific enough to study seasonal influenza because they include all influenza subtypes and lineages, as well as other seasonal respiratory pathogens that can also have a significant prevalence^45^. Hence, even jurisdictions with a strong surveillance system may not have more than 20 years’ worth of genetically characterized data on seasonal influenza. Moreover, considering that the current three main influenza (sub)types (A/H1N1, A/H3N2 and B) do not circulate every season, we can expect to have about 10 seasons with a sufficient sample size for an insightful statistical analysis.

All the challenges described above apply to our study. Our definitions of epitopes were simply based on previous studies and did not consider specific qualitative properties of genetic mutations. The volume of genetic data sourced from GISAID (after restricting the data set to Canadian sequences) and the volume of epidemiological data were limited to a few hundred sequences and to no more than eight seasons for each of the main seasonal influenza virus types and subtypes, hampering our statistical analysis of the relationship between epidemiological severity and genetic drift.

Unlike previous studies^9,10^, our statistical analysis could not identify an effect of either the genetic drift of seasonal influenza or vaccine effectiveness on the observed epidemiological severity of seasonal influenza epidemics in Canada. This “negative result” could be explained by an actual lack of, or a very small, combined effect of the genetic drift and vaccine effectiveness on the severity of seasonal influenza epidemics. In that case, other factors, like the effect of co-circulation of other respiratory pathogens, may have more importance in explaining the epidemiological severity of seasonal influenzas. Another explanation could be that our methodological approach was not correctly designed. Indeed, our study has several limitations.

We observed that the mean inter-seasonal antigenic drift tended to be higher in influenza B compared to influenza A viruses. This is due to pairwise comparison between sequences of the B/Victoria and B/Yamagata lineages, which are antigenically distinct and co-circulate in the population^46^. Although the two lineages have diverged genetically, this distinction is not made in the epidemiological data, resulting in larger mean antigenic distances between seasons. Previous studies have shown that HA is more genetically similar across influenza B lineages than between influenza A subtypes^47^, and provide evidence of cross-lineage protection from vaccines^48^. However, this does not necessarily contradict our findings. Cross-protective antibodies often (though not always) recognize conserved epitopes^49^, while our analysis focused on genetic changes in canonical antigenic sites, which are more susceptible to mutation. In other words, our decision to utilize a sequence-based estimate of antigenic distance may explain these seemingly opposing points, and larger antigenic distances between circulating influenza B strains may not necessarily correspond to an increased burden of disease.

Our use of genetic data was simplified. All influenza sequences were sourced from GISAID. Although this is one of the main sources of genetic data (Influenza Research Database (IRD) being another one), the uploaded sequences may not be representative of all influenza viruses circulating in Canada. We calculated the inter-season genetic distance using the Hamming distance, giving equal weight to each amino acid change, ignoring mutations that may have an outsized impact on antigenicity. We also considered only the hemagglutinin protein when assessing the genetic distance but ignored the neuraminidase (NA) protein. Recognition of the NA protein also plays a role in immune response to influenza infection^50^, and antigenic drift in NA is a major driver of B/Yamagata epidemics^46^.

Although we could not establish a significant statistical effect of vaccine effectiveness on the severity index, this does not mean that influenza vaccines are ineffective. In our study, the focal variable was the inter-season genetic distance of influenza viruses. Vaccine effectiveness was introduced in our regression as a potential effect modifier. Many studies have shown the value of vaccination against seasonal influenza in preventing serious outcomes^51–55^. In addition, any conclusions about the role of vaccine effectiveness on epidemic severity would need to include vaccine coverage, which, even if available, may be misleading given the ecological nature of our analysis. Since we primarily worked with aggregate measures of seasonal influenza severity, we often lacked data on individual vaccination status. Although we assumed vaccine coverage to be constant throughout the study period, there is no way to ascertain the percentage of vaccinated or unvaccinated individuals among the lab-confirmed cases or influenza-associated hospitalizations and deaths reported in a given season. Hence, absence of a significant statistical effect from vaccination in our simplified study cannot be extrapolated to determine the impact of influenza vaccination.

There are also limitations on the epidemiological side of our study. We ignored co-infection and co-circulation of other seasonal respiratory pathogens to keep the complexity of this study manageable, but there is some evidence that interaction with other pathogens at the individual and population levels may be relevant to understand the drivers of seasonal influenza severity^39,56^. As a further simplification, we did not consider environmental factors despite some evidence they may be implicated in influenza dynamics^57,58^. Furthermore, we ignored age-related heterogeneity in vaccine coverage and effectiveness, as well as in the severity of influenza infections. Vaccine uptake tends to be higher in older individuals, while vaccine effectiveness may vary by age due to immunosenescence and immune imprinting^59^. Related to this, the distribution of influenza types and subtypes amongst predominant circulating strains in a given flu season may affect population susceptibility in an age-dependent manner. H3N2 infections tend to more severely impact older adults compared to younger age groups^60^ (with the opposite being true of H1N1^61^), and influenza B may also be detected less frequently in the former than in the latter^62^. Due to the lack of age-specific data in some of the measures used in our regression model, we were not able to conduct stratified analyses to capture the nuances that might exist as a result of age. Similarly, a lack of genetic detail linked to epidemiological data prevented a finer analysis. For example, two antigenically distinct H3N2 clades (3C.2a and 3C.3a) have been circulating since 2014^63^ and should probably have been treated separately in our analysis; however, the clade is rarely identified in reported cases. There is also no distinction made between B/Victoria and B/Yamagata in the weekly surveillance data. As a result, we were unable to separate antigenically distinct clades or lineages when calculating antigenic distances, thereby limiting the conclusions we can draw about the role of antigenic drift in determining seasonal severity.

It may also be possible that there are no clearly observable variables that contribute to the severity of seasonal influenza epidemics. Instead, there may be a myriad of small, practically undetectable effects that add up together and ultimately determine the severity of an influenza season^64^. This would hamper our ability to project the severity of an upcoming influenza season. More advanced computational and statistical techniques (e.g., machine learning) could be tried, but the issue of data volume should be addressed first.

In any case, our understanding of the potential factors that can affect the severity of seasonal influenza epidemics could be vastly improved by enhancing several areas. Our study has highlighted the need for more systematically subtyping and recording influenza infections detected via traditional surveillance, as well as expanding this surveillance to populations other than severe cases in order to have a clearer picture of the relative severity of seasonal influenza epidemics. With more detailed epidemiological data, the mutations of the genome of a given influenza subtype or lineage can be scrutinized through the lens of its epidemiological footprints. This would allow us to gain a better qualitative understanding of specific mutations on immunity and/or virulence. Finally, studies of interactions between influenza viruses and other respiratory pathogens at the patient level could shed light on the importance of co-circulation for transmission dynamics of influenza at the population level. The COVID-19/SARS-CoV-2 pandemic has demonstrated that intense epidemiological surveillance and fundamental research can be productive to understanding the dynamics of various lineages (e.g., their immunogenicity, transmission, virulence) and potentially controlling epidemic trajectories. Although the level of investments needed to perform such surveillance and research for SARS-CoV-2 has already eclipsed that of decades of influenza surveillance, it is probably not sustainable for seasonal influenza. Nonetheless, it suggests that our understanding of the sources of seasonal influenza severity could be increased, given enough resources.

## Conclusion

This study could not establish that the antigenic drift of common influenza viruses has an effect on the epidemiological severity of seasonal influenza epidemics in Canada. To identify the potential factors driving seasonal influenza severity, future studies may need to focus on variables not considered here and/or accumulate more data.

## Supplementary Information

Supplementary Information 1.

Supplementary Information 2.

Supplementary Information 3.

Supplementary Information 4.

Supplementary Information 5.

Supplementary Information 6.

Supplementary Information 7.

Supplementary Information 8.

## Data Availability

The datasets generated during and/or analysed during the current study are available from the corresponding author on reasonable request.
