# Publisher Correction: Antigenic drift and epidemiological severity of seasonal influenza in Canada

**DOI:** 10.1038/s41598-024-53476-4

**Published:** 2024-02-05

**Authors:** Zishu Chen, Christina Bancej, Liza Lee, David Champredon

**Affiliations:** 1 National Microbiology Laboratory, Public Health Risk Sciences Division, Public Health Agency of Canada, Guelph, ON Canada; 2 Surveillance and Epidemiology Division, Centre for Immunization and Respiratory Infectious Disease, Public Health Agency of Canada, Ottawa, ON Canada; 3 Dalla Lana School of Public Health, University of Toronto, Toronto, ON Canada

Correction to: *Scientific Reports* 10.1038/s41598-022-19996-7, published online 17 September 2022

The original version of this Article contained an error in the Methods, under the subheading ‘Severity index’, where the equation for the severity index $S$ was a duplicate of the linear regression stated under the subheading ‘Statistical analysis’.

As a result, under the subheading ‘Severity index’,

$$ S \sim \beta_{{\left\{ 1 \right\}}} *a + \beta_{{\left\{ 2 \right\}}} *v + \beta_{{\left\{ 3 \right\}}} *a*v, $$

now reads:

$$S = ( P + H + D + R)/n,$$

The original Article has been corrected.

