# Supplementary material for: Antigenic drift and epidemiological severity of seasonal influenza in Canada
Source: Sci Rep. 2022 Sep 17;12:15625. doi: 10.1038/s41598-022-19996-7 (PMC9482630; doi:10.1038/s41598-022-19996-7)
Supplement: Supplementary file 2 — Supplementary Information 2. [file 41598_2022_19996_MOESM2_ESM.docx]

Supplementary Materials

## Antigenic site residue positions

*“Narrow” definition by antigenic site*

| **Subtype** | **Antigenic Site** | **Residue Positions*** |
| --- | --- | --- |
| H1N1 [1,2] | Sa | 141, 142, 170-174, 176-181 |
|  | Sb | 201-212 |
|  | Ca1 | 183-187, 220-222, 252-254 |
|  | Ca2 | 154-159, 238, 239 |
|  | Cb | 87-92 |
| H3N2 [1,2] | A | 138, 140, 142, 146-149, 151, 153, 154, 156, 158-162, 166, 168, 184 |
|  | B | 144, 145, 171-176, 179-181, 202-206, 208-210, 212-214 |
|  | C | 60-64, 66, 67, 69, 70, 289, 291, 292, 294-296, 310, 313, 315, 316, 320, 321, 323-328 |
|  | D | 112, 118, 119, 133, 137, 183, 186-193, 195, 198, 217, 219, 223-225, 228-235, 242-246, 254, 256, 258, 260, 262-264 |
|  | E | 73, 75, 78, 79, 83, 91, 94, 96-99, 102-104, 107, 108, 110, 125, 276-278, 281 |
| B [2–4] | 120 loop | 63, 71, 88-94, 131-152, 197-199 |
|  | 150 loop | 156-165 |
|  | 160 loop | 177-185 |
|  | 190 helix | 212-220 |
|  | 230 region | 244-259 |

* Numbering starts at the first methionine (M) of the HA protein sequence. Signal peptides (residues 1-17 for H1N1, 1-16 for H3N2 and 1-15 for B) were excluded from analysis.

*Site positions by definition*

Initial analyses were run using the “Narrow”, “Broad” and “Full” (i.e., full HA1 + HA2) definitions. Sensitivity analyses were then run using the “Broad1”, “Misc1” and “Misc2” (when applicable) definitions. Misc1 and Misc2 were defined by selecting residue positions that have been found to be potentially more antigenically relevant or that have been identified as epitopes in studies of antibody escape mutations, based on a non-exhaustive search of the literature.

| **Subtype** | **Definition** | **Residue Positions*** |
| --- | --- | --- |
| H1N1 | Narrow | 87-92, 141, 142, 154-159, 170-174, 176-181, 183-187, 201-212, 220-222, 238, 239, 252-254 |
|  | Broad (narrow + 3AA buffer) | 84-95, 138-145, 151-162, 167-190, 198-215, 217-225, 235-242, 249-257 |
|  | Broad1 (narrow + 1AA buffer) | 86-93, 140-143, 153-160, 169-188, 200-213, 219-223, 237-240, 251-255 |
|  | Full HA1** | 18-344 |
|  | Full HA1 + HA2 | 18-566 |
|  | Misc1 (selected sites) [5–9] | 136, 142, 144, 147, 148, 158, 169-173, 180, 200, 204, 241 |
| H3N2 | Narrow | 60-64, 66, 67, 69, 70, 73, 75, 78, 79, 83, 91, 94, 96-99, 102-104, 107, 108, 110, 112, 118, 119, 125, 133, 137, 138, 140, 142, 144-149, 151, 153, 154, 156, 158-162, 166, 168, 171-176, 179-181, 183, 184, 186-193, 195, 198, 202-206, 208-210, 212-214, 217, 219, 223-225, 228-235, 242-246, 254, 256, 258, 260, 262-264, 276-278, 281, 289, 291, 292, 294-296, 310, 313, 315, 316, 320, 321, 323-328 |
|  | Broad (narrow + 3AA buffer) | 57-86, 88-128, 130-249, 251-267, 273-284, 286-299, 307-331 |
|  | Broad1 (narrow + 1AA buffer) | 59-80, 82-84, 90-113, 117-120, 124-126, 132-134, 136-163, 165-199, 201-220, 222-236, 241-247, 253-265, 275-282, 288-297, 309-317, 319-329 |
|  | Full HA1** | 17-345 |
|  | Full HA1 + HA2 | 17-566 |
|  | Misc1 (selected sites) [10] | 41, 66, 69, 70, 78, 91, 98, 99, 138, 140, 147, 149, 153, 159-162, 171, 172, 174, 176, 180, 188, 190, 204-206, 209, 212, 213, 217, 218, 223, 229, 233, 238, 241, 246, 260, 276, 278, 292, 294 |
|  | Misc2 (selected sites) [5,11] | 161, 171, 172, 174, 175, 205, 206, 209 |
| B | Narrow | 63, 71, 88-94, 131-152, 156-165, 177-185, 197-199, 212-220, 244-259 |
|  | Broad (narrow + 3AA buffer) | 60-66, 68-74, 85-97, 128-168, 174-188, 194-202, 209-223, 241-262 |
|  | Broad1 (narrow + 1AA buffer) | 62-64, 70-72, 87-95, 130-153, 155-166, 176-186, 196-200, 211-221, 243-260 |
|  | Full HA1** | 16-360 |
|  | Full HA1 + HA2 | 16-585 |
|  | Misc1 (selected sites) [5] | 180, 181 |

AA = amino acid

* Numbering starts at the first methionine (M) of the HA protein sequence. Signal peptides (residues 1-17 for H1N1, 1-16 for H3N2 and 1-15 for B) were excluded from analysis.

** In a preliminary regression analysis, the full HA1 and full HA1+HA2 definitions yielded similar results. Consequently, the full HA1 definition was excluded from further analyses, and only the full HA sequence (i.e., HA1+HA2) was considered.

References:

1. Skowronski, D. M. *et al.* Low 2012–13 Influenza Vaccine Effectiveness Associated with Mutation in the Egg-Adapted H3N2 Vaccine Strain Not Antigenic Drift in Circulating Viruses. *PLOS ONE* **9**, e92153 (2014).

2. Skowronski, D. M. *et al.* Integrated Sentinel Surveillance Linking Genetic, Antigenic, and Epidemiologic Monitoring of Influenza Vaccine-Virus Relatedness and Effectiveness During the 2013–2014 Influenza Season. *The Journal of Infectious Diseases* **212**, 726–739 (2015).

3. Wang, Q., Cheng, F., Lu, M., Tian, X. & Ma, J. Crystal Structure of Unliganded Influenza B Virus Hemagglutinin. *Journal of Virology* **82**, 3011–3020 (2008).

4. Ni, F., Kondrashkina, E. & Wang, Q. Structural basis for the divergent evolution of influenza B virus hemagglutinin. *Virology* **446**, 112–122 (2013).

5. Koel, B. F. *et al.* Substitutions near the receptor binding site determine major antigenic change during influenza virus evolution. *Science (1979)* **342**, 976–979 (2013).

6. Koel, B. F. *et al.* Identification of Amino Acid Substitutions Supporting Antigenic Change of Influenza A(H1N1)pdm09 Viruses. *Journal of Virology* **89**, 3763–3775 (2015).

7. Rudneva, I. *et al.* Escape mutants of pandemic influenza A/H1N1 2009 virus: variations in antigenic specificity and receptor affinity of the hemagglutinin. *Virus Res* **166**, 61–67 (2012).

8. Manicassamy, B. *et al.* Protection of Mice against Lethal Challenge with 2009 H1N1 Influenza A Virus by 1918-Like and Classical Swine H1N1 Based Vaccines. *PLOS Pathogens* **6**, e1000745 (2010).

9. O’Donnell, C. D. *et al.* Antibody pressure by a human monoclonal antibody targeting the 2009 pandemic H1N1 virus hemagglutinin drives the emergence of a virus with increased virulence in mice. *mBio* **3**, (2012).

10. Smith, D. J. *et al.* Mapping the antigenic and genetic evolution of influenza virus. *Science (1979)* **305**, 371–376 (2004).

11. Beer, K. *et al.* Characterization of neutralizing epitopes in antigenic site B of recently circulating influenza A(H3N2) viruses. *The Journal of General Virology* **99**, 1001 (2018).
