# Supplementary material for: Antigenic drift and epidemiological severity of seasonal influenza in Canada
Source: Sci Rep. 2022 Sep 17;12:15625. doi: 10.1038/s41598-022-19996-7 (PMC9482630; doi:10.1038/s41598-022-19996-7)

Historical time series of laboratory-confirmed cases of influenza in Canada by type and subtype. Source: PHAC.


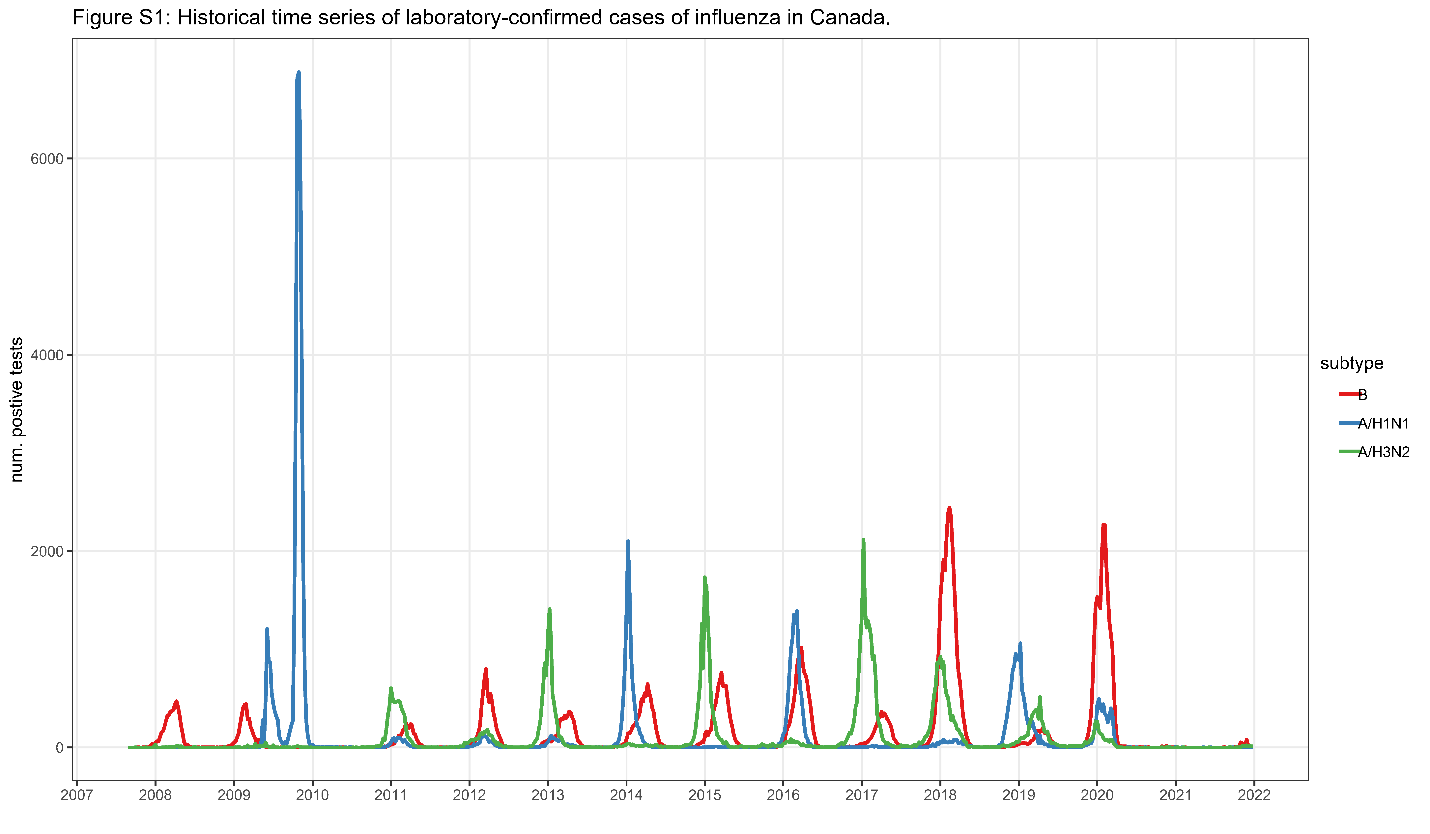

Supplement: Supplementary file 3 — Supplementary Information 3. [file 41598_2022_19996_MOESM3_ESM.docx]
