# Supplementary material for: Antigenic drift and epidemiological severity of seasonal influenza in Canada
Source: Sci Rep. 2022 Sep 17;12:15625. doi: 10.1038/s41598-022-19996-7 (PMC9482630; doi:10.1038/s41598-022-19996-7)

**Figure S6**: Same as Figure 3 in the main text, but with a lag of two seasons (instead of one) between the severity index and the antigenic distance.


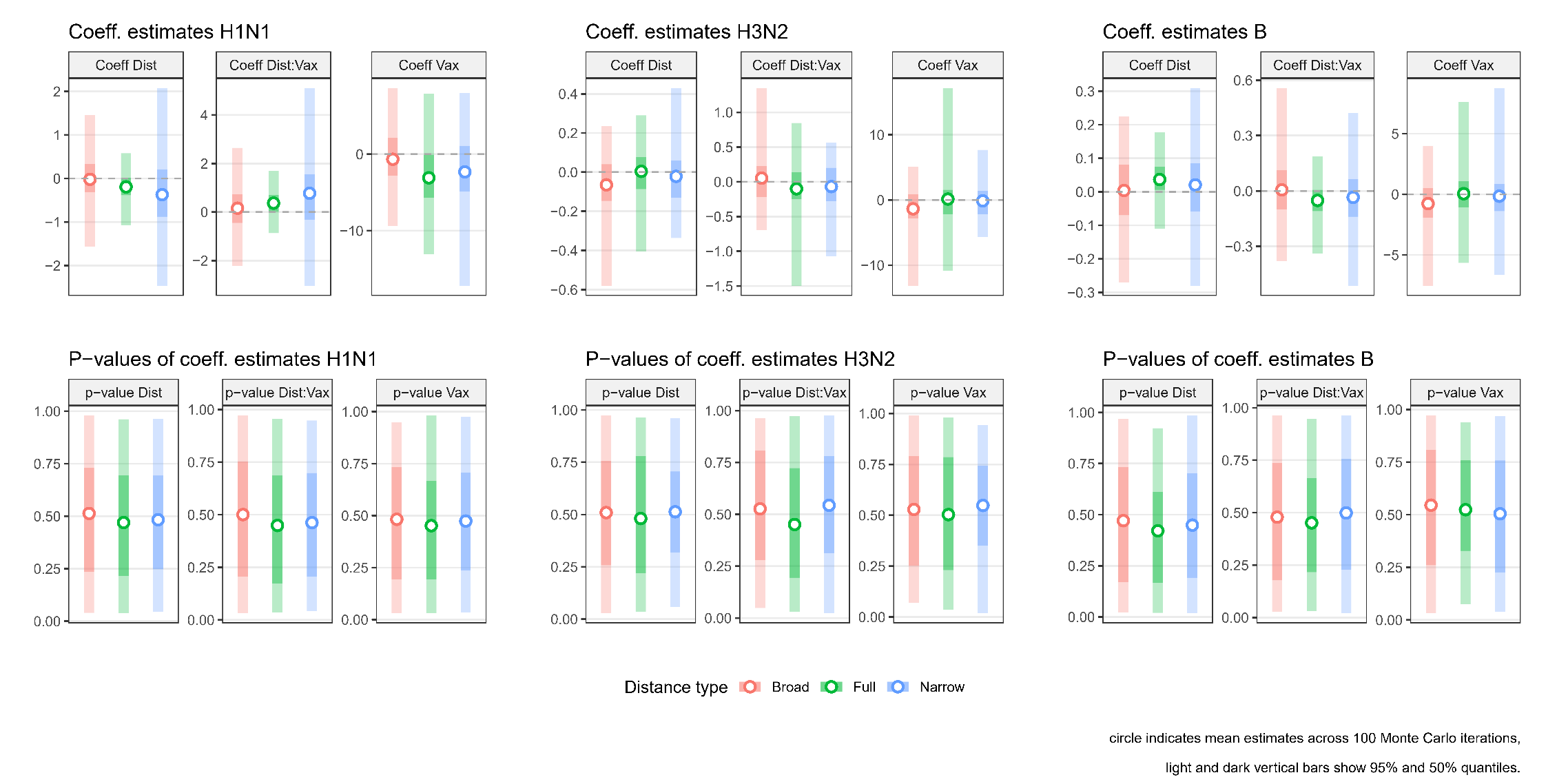

Supplement: Supplementary file 6 — Supplementary Information 6. [file 41598_2022_19996_MOESM6_ESM.docx]
