# Supplementary material for: Antigenic drift and epidemiological severity of seasonal influenza in Canada
Source: Sci Rep. 2022 Sep 17;12:15625. doi: 10.1038/s41598-022-19996-7 (PMC9482630; doi:10.1038/s41598-022-19996-7)

# Supplementary material file S7: sensitivity analysis of the severity index definition

**Figure S7a**: Same as Figure 3 in the main text, but with the severity index collapsed to **pediatric hospitalization rate** associated with influenza.


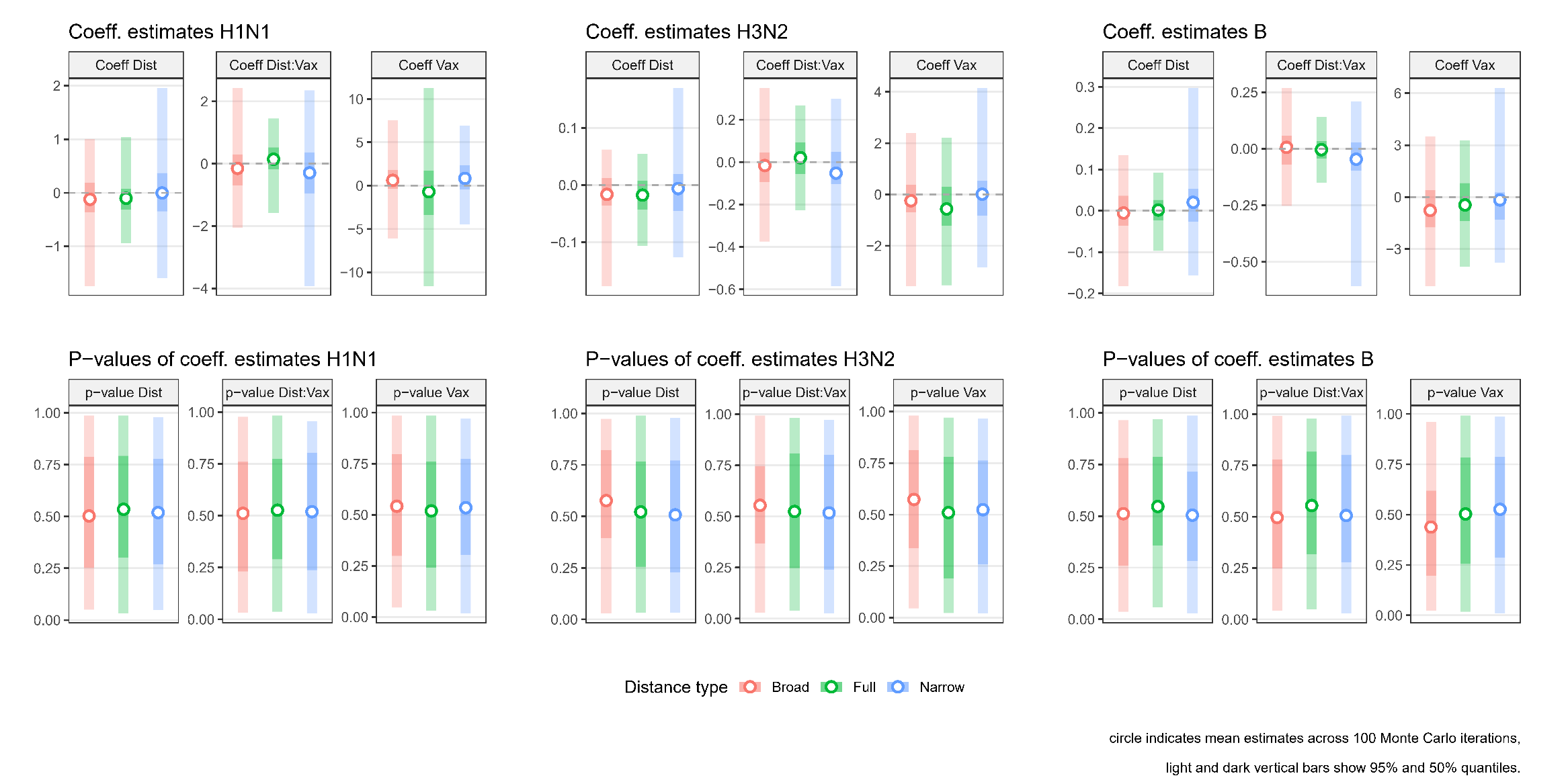


**Figure S7b**: Same as Figure 3 in the main text, but with the severity index collapsed to **mortality rate** associated with influenza and pneumonia.


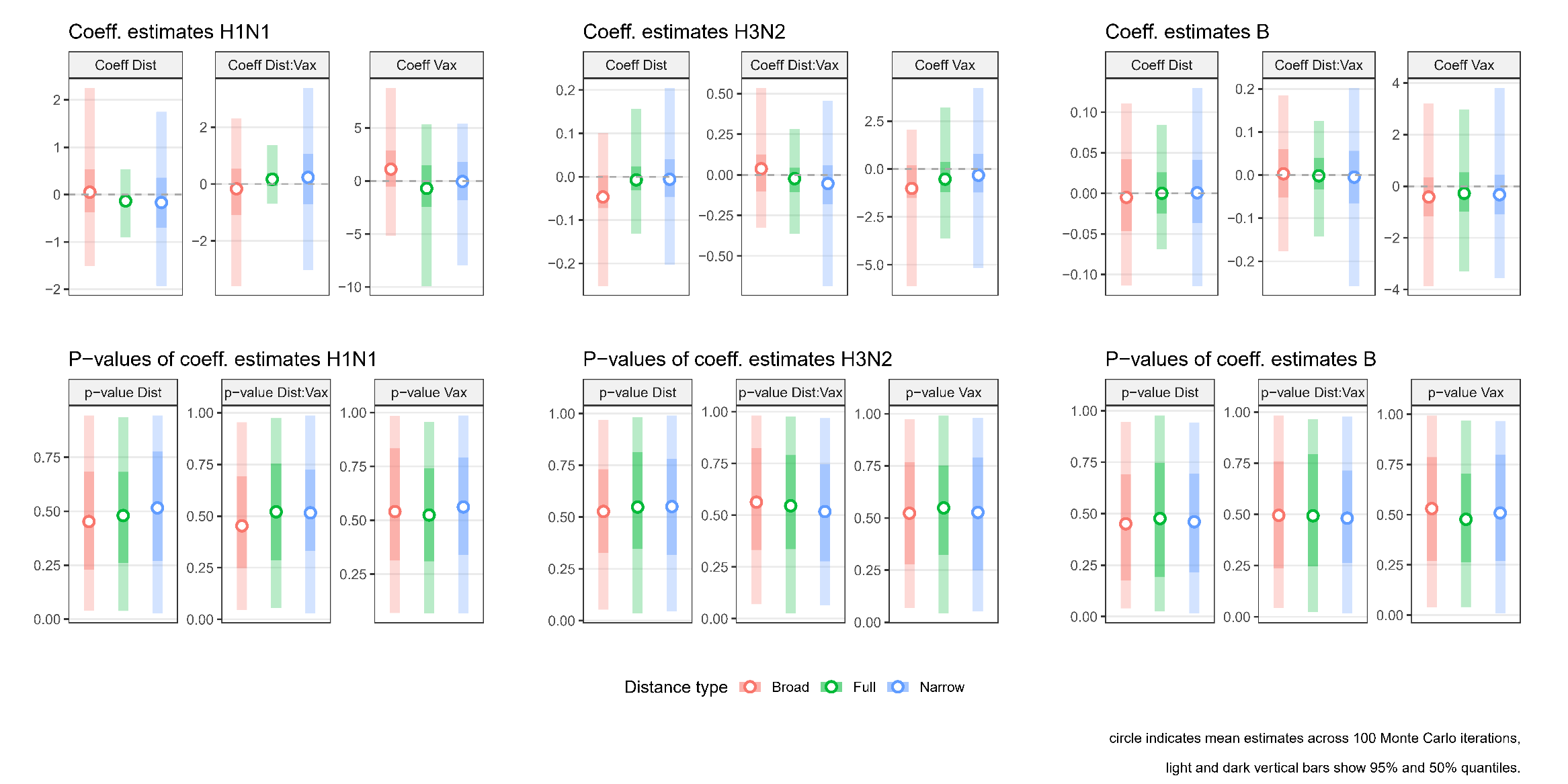


**Figure S7c**: Same as Figure 3 in the main text, but with the severity index collapsed to the **peak positivity rate** associated with reported influenza cases.


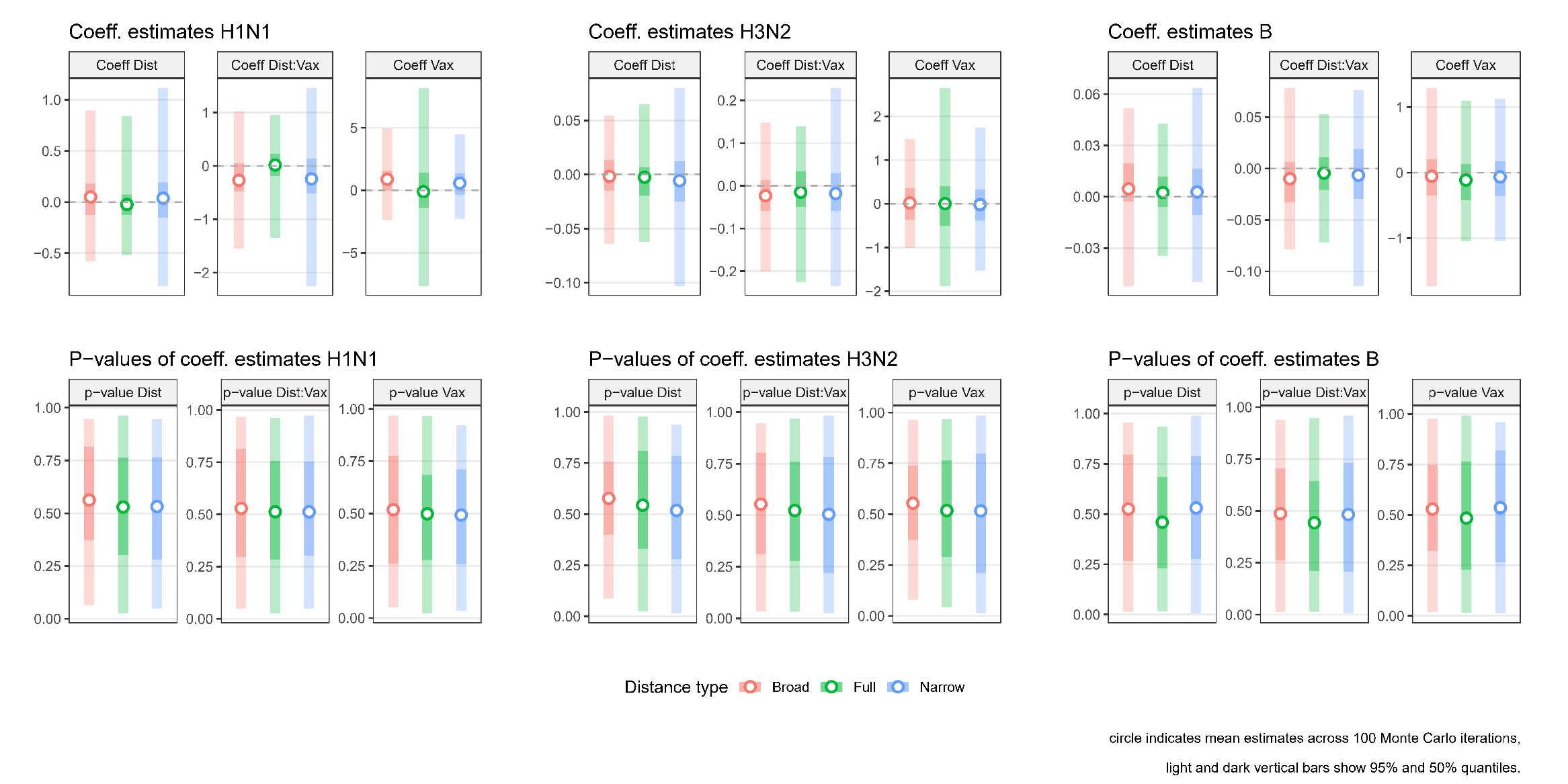


**Figure S7d**: Same as Figure 3 in the main text, but with the severity index collapsed to **basic reproduction number** of reported influenza cases.


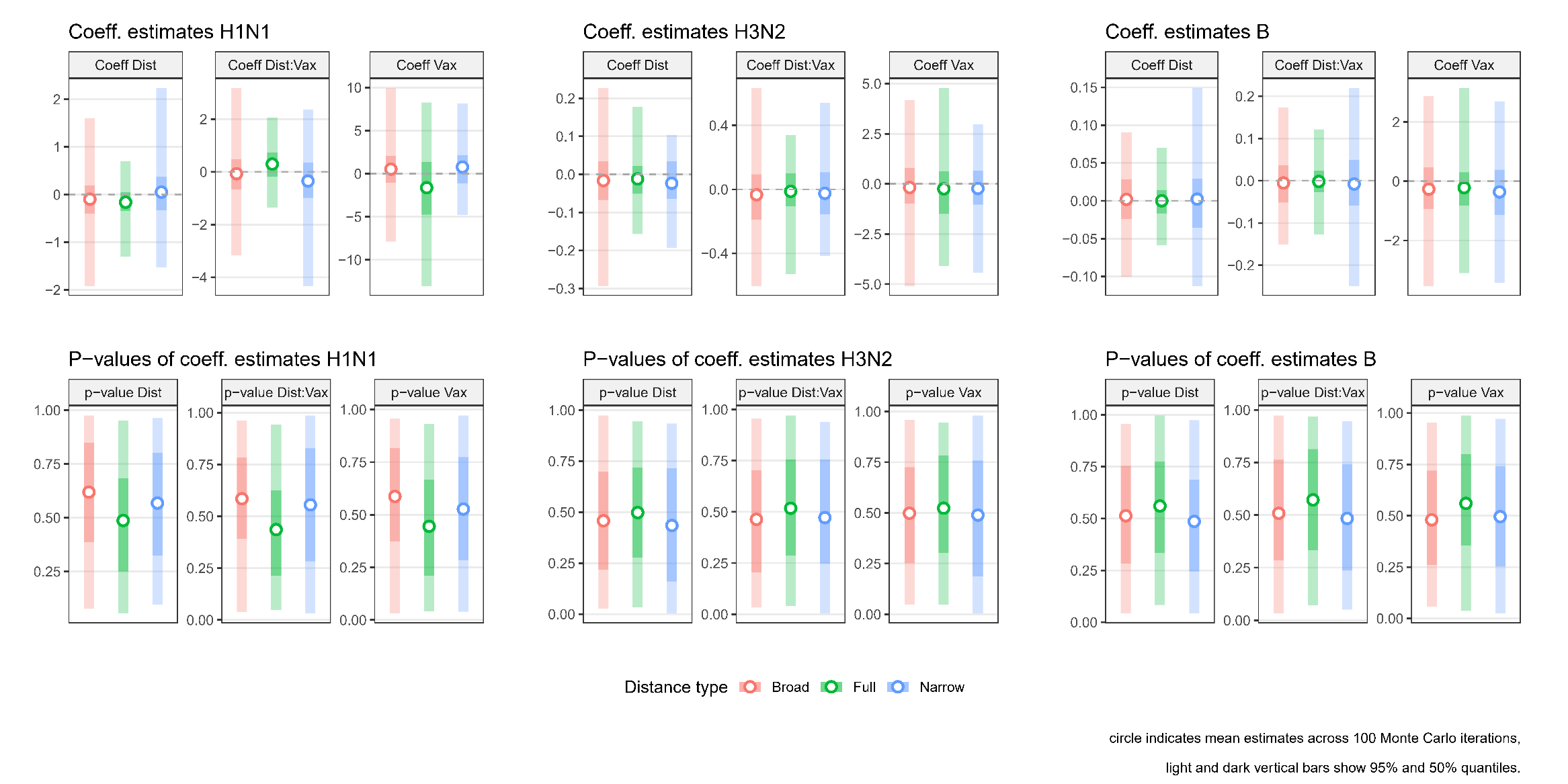

Supplement: Supplementary file 7 — Supplementary Information 7. [file 41598_2022_19996_MOESM7_ESM.docx]
