# Supplementary material for: Antigenic drift and epidemiological severity of seasonal influenza in Canada
Source: Sci Rep. 2022 Sep 17;12:15625. doi: 10.1038/s41598-022-19996-7 (PMC9482630; doi:10.1038/s41598-022-19996-7)

**Figure S8:** Same as Figure 3 in the main text, with various definitions of epitope sites. See table S2 for the definition of the epitope sites “Narrow”, “Broad”, “Broad1”, “Misc1”, “Misc2” and “Full”.


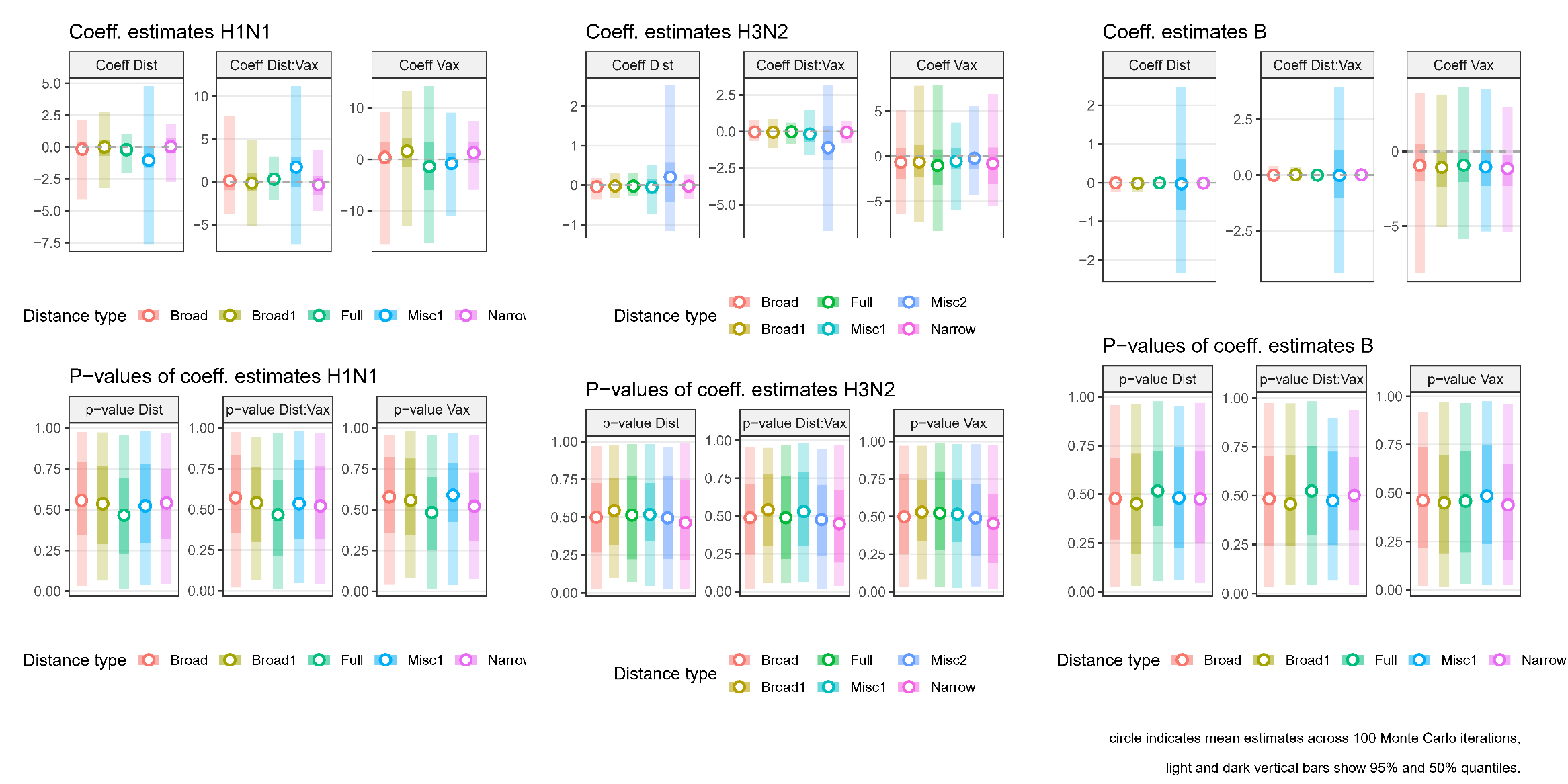

Supplement: Supplementary file 8 — Supplementary Information 8. [file 41598_2022_19996_MOESM8_ESM.docx]
